# High incidence of surgical site infection may be related to suboptimal case selection for non-selective arterial embolization during resuscitation of patients with pelvic fractures: a retrospective study

**DOI:** 10.1186/s12891-020-03372-5

**Published:** 2020-05-30

**Authors:** Chih-Yang Lai, I-Chuan Tseng, Chun-Yi Su, Yung-Heng Hsu, Ying-Chao Chou, Huan-Wu Chen, Yi-Hsun Yu

**Affiliations:** 1 Department of Orthopedic Surgery, Musculoskeletal Research Center, Chang Gung Memorial Hospital, Linkou branch, and Chang Gung University 33302, Tao-Yuan, Taiwan. 5, Fu-Hsin St. Kweishan, 33302 Tao-Yuan, Taiwan; 2 Department of Orthopedic Surgery, Chang Gung Memorial Hospital, Keelung Branch, and Chang Gung University, Keelung City, Taiwan; 3 Department of Medical Imaging & Intervention, Chang Gung Memorial Hospital, Linkou Branch, and Chang Gung University, Taoyuan City, Taiwan

**Keywords:** Trauma of pelvis, Infection, Major trauma management, Resuscitation, And critical care

## Abstract

**Background:**

In most institutions, arterial embolization (AE) remains a standard procedure to achieve hemostasis during the resuscitation of patients with pelvic fractures. However, the actual benefits of AE are controversial. In this study, we aimed to explore AE-related outcomes following resuscitation at our center and to assess the predictive value of contrast extravasation (CE) during computed tomography (CT) for patients with hemodynamically unstable closed pelvic fractures.

**Methods:**

We retrospectively reviewed data from patients who were treated for closed pelvic fractures at a single center between 2014 and 2017. Data regarding the AE and clinical parameters were analyzed to determine whether poor outcomes could be predicted.

**Results:**

During the study period, 545 patients were treated for closed pelvic fractures, including 131 patients who underwent angiography and 129 patients who underwent AE. Nonselective bilateral internal iliac artery embolization (nBIIAE) was the major AE strategy (74%). Relative to the non-AE group, the AE group had higher values for injury severity score, shock at hospital arrival, and unstable fracture patterns. The AE group was also more likely to require osteosynthesis and develop surgical site infections (SSIs). Fourteen patients (10.9%) experienced late complications following the AE intervention, including 3 men who had impotence at the 12-month follow-up visit and 11 patients who developed SSIs after undergoing AE and osteosynthesis (incidence of SSI: 11/75 patients, 14.7%). Nine of the 11 patients who developed SSI after AE had undergone nBIIAE. The positive predictive value of CE during CT was 29.6%, with a negative predictive value of 91.3%. Relative to patients with identifiable CE, patients without identifiable CE during CT had a higher mortality rate (30.0% vs. 11.0%, *p* = 0.03).

**Conclusion:**

Performing AE for pelvic fracture-related hemorrhage may not be best practice for patients with no CE detected during CT or for unstable patients who do not respond to resuscitation after exclusion of other sources of hemorrhage. Given the high incidence of SSI following nBIIAE, this procedure should be selected with care. Given their high mortality rate, patients without CE during imaging might be considered for other hemostasis procedures, such as preperitoneal pelvic packing.

## Background

Pelvic fractures can often result in potentially lethal hemorrhage and hypotension [1, 2]. Thus, hemodynamically unstable patients with pelvic fractures present a major challenge for acute care and orthopedic surgeons, and such patients are best managed by a multi-disciplinary team [3]. However, the appropriate acute management of hemodynamically unstable patients with pelvic fractures remains controversial. Arterial embolization (AE) and preperitoneal pelvic packing (PPP) are common procedures in this setting, although there is no conclusive evidence for the superiority of either procedure [2, 4, 5].

Some reports have described AE as an effective procedure for managing pelvic fracture-related retroperitoneal arterial hemorrhage [6, 7]. AE is a classical treatment and remains a standard hemostatic procedure in many institutions [7–9], including our institution, based on reports regarding its efficacy during the management of pelvic fracture-related hemorrhage [8, 10]. However, AE is associated with various complications, including inadequate hemostasis, gluteal muscle necrosis, and surgical site infection (SSI) [11, 12]. Therefore, as an observation of contrast extravasation (CE) during computed tomography (CT) [13] is unreliable as a sole basis for selecting AE, additional information is needed to help determine when AE can help provide optimal outcomes. This study aimed to explore AE-related outcomes following resuscitation at our center and to assess the predictive value of CE during CT for patients with hemodynamically unstable closed pelvic fractures.

## Methods

At our institution, AE is a standard procedure for pelvic fracture-related hemorrhage and is performed when CE is detected during contrast-enhanced CT or for unstable patients who do not respond to resuscitation after the exclusion of other sources of hemorrhage. Angiography is initially performed to evaluate the abdominal aorta, lumbar arteries, bilateral common iliac arteries, bilateral external iliac arteries, and bilateral internal iliac arteries. When arterial bleeding is detected or suspected, the first embolization material is generally gelfoam (SPONGOSTAN, Ethicon Inc., Belgium), and bleeding is re-checked 5 min later. If an aneurysm persists or CE recurs, permanent embolization is performed using metal coils (Tornado Embolization Coils, Cook Inc., USA or VortX-18 Coils, Boston Scientific, USA). Selective or non-selective embolization is performed at the discretion of the interventional radiologist. After any AE, the patient is admitted to the intensive care unit or the ward, depending on the clinical requirements. Osteosynthesis is then performed for pelvic ring injuries after the patient’s clinical condition has stabilized. During the study period, resuscitative endovascular balloon occlusion of the aorta was not available for managing these patients at our center.

We retrospectively reviewed trauma registry data, including clinical and imaging records, from all cases of pelvic fracture that were treated at our center between January 2014 and December 2017. The study’s retrospective protocol was approved by our institutional review board (IRB: 201802139B0). Patients were excluded if they had an isolated acetabular fracture, were dead upon arrival, were diagnosed with a pelvic fracture without imaging, or had undergone AE as a hemostatic procedure targeting non-pelvic regions. All patients with pelvic fractures were resuscitated and managed according to our established protocol, which was based on the Advanced Trauma Life Support guidelines.

The patients’ demographic characteristics, injury severity score (ISS), fracture patterns, AE details, and AE-related complications were recorded. Complications were defined as adverse responses or unexpected conditions that were likely related to the AE procedure. We defined an SSI as a post-osteosynthesis wound complication, which yielded a positive bacterial culture. Information regarding impotence in men was self-reported at the 12-month follow-up visit.

Data were analyzed using SPSS software (version 18.0; SPSS Inc., Chicago, IL). Continuous variables were compared using the t-test, and categorical variables were compared using the chi-squared test and Fisher’s exact test. Predictive analyses were performed to determine whether the presence of CE during CT could be used to predict clinical outcomes.

## Results

During the 48-month study period, we identified 545 patients with closed pelvic fractures who underwent resuscitation and treatment (Table 1). Traffic accident was the most common injury mechanism (70.2%). Initial clinical assessments revealed that 112 patients (20.6%) were in shock at the initial presentation and required resuscitation and blood transfusions. After resuscitation, 49 patients (43.8%) were identified as responders, and the remaining patients did not initially respond to resuscitation. The CT assessments revealed that 390 patients (71.5%) had partially stable or unstable ring patterns. Angiography was performed for 131 patients (24%) who were unresponsive to resuscitation or who had CE during the CT examination. There were no complications during or immediately after the angiography procedure, and AE was performed for 129 of the 131 patients (98.5%) who underwent angiography (Table 2).

**Table 1:** Demographic characteristics of patients with pelvic fracture during 2014–2017 at our institution

Total number of patients	545
Mean age, years	46.2 ± 21.6
Sex, n (%)	
Male	267 (49.0%)
Female	278 (51.0%)
Trauma mechanism, n (%)	
Traffic accident	383 (70.2%)
Falling from height	145 (26.6%)
Other	17 (3.2%)
Mean Injury Severity Score	17.4 ± 12.2
Shock on arrival, *n* (%)	112 (20.6%)
Fracture classification, n (%) †	
Stable ring	277 (50.8%)
Partially stable ring	113 (20.7%)
Unstable ring	155 (28.5%)
Imaging used to evaluate arterial bleeding, *n* (%)	
Computed tomography	545 (100%)
Angiography	131 (24.0%)
Arterial embolization, *n* (%)	
No	416 (76.3%)
Yes	129 (23.7%)
Osteosynthesis for pelvic fracture, *n* (%)	211 (38.7%)
Mortality, *n* (%)	27 (4.9%)

† The classification of the pelvic fracture was based on the AO/OTA classification (2018 revision)

**Table 2:** Characteristics of the patients who underwent arterial embolization

Total patients, n	129
Mean time from order to procedure, min	63.8 ± 44.7
Shock on arrival, *n* (%)	90 (69.7%)
Non-response to resuscitation, *n* (%)	47 (36.4%)
Contrast extravasation, n (%)	
During CT	107 (82.9%)
During angiography	34 (26.3%)
Location of AE, n (%)	
BIIA	97 (74.0%)
RIIA	17 (12.9%)
LIIA	15 (11.8%)
Material used for embolization, n (%)	
Gelfoam	118 (91.4%)
Metal coils	11 (8.6%)
Mean blood transfusion, mL	2388.1 ± 2633.6
Osteosynthesis surgery, n (%)	75 (58.1%)
AE-related complications, n (%)	
Impotence in men	3
Surgical site infection	11

*CT* computed tomography, *AE* arterial embolization, *BIIA* bilateral internal iliac arteries, *RIIA* right internal iliac artery, *LIIA* left internal iliac artery

**Fig. 1:**
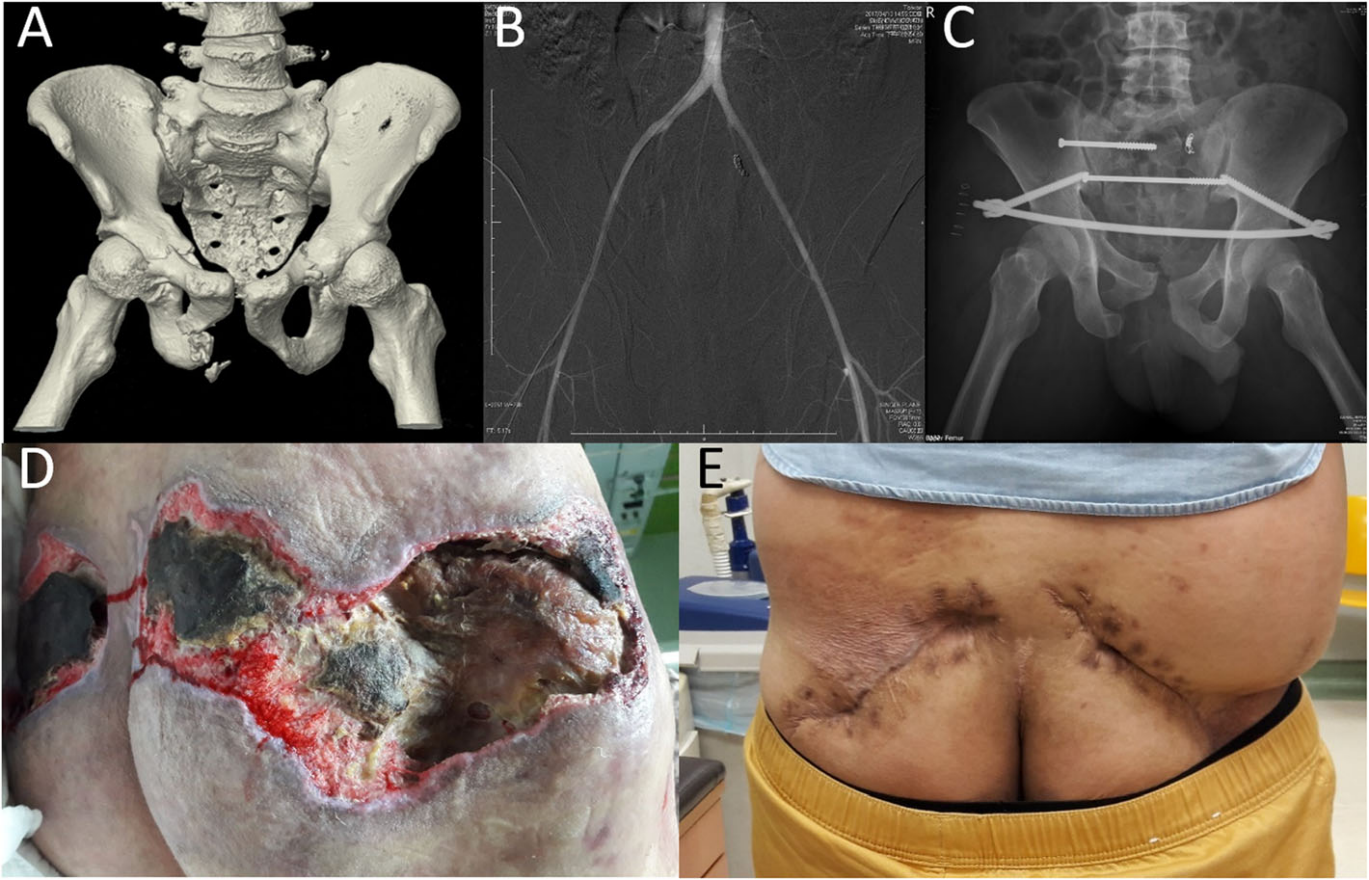
A case with surgical site infection following trans-arterial embolization and osteosynthesis for pelvic ring injury. **a** Computed tomography revealed an AO/OTA B3.1 pelvic ring injury. **b** Bilateral internal iliac artery trans-arterial embolization was performed using gelfoam (right side) and metal coil (left side). **c** Radiographic findings after the osteosynthesis. **d** Significant bilateral gluteus maximus necrosis was observed at 1 month after the trans-arterial embolization. **e** Final appearance after treatment for the soft-tissue defect

The average time from the order to the angiography procedure was 63.8 ± 44.7 min. Ninety-seven patients (74.0%) underwent non-selective bilateral internal iliac artery embolization (nBIIAE) using gelfoam as the filling material. Only 11 of the 129 patients (8.5%) underwent AE using metal coils. Fourteen patients (10.9%) experienced late complications after the AE intervention, including 3 men who had impotence at the 12-month follow-up visit and 11 patients who developed SSIs after the AE and osteosynthesis (Fig. 1). The incidence of SSIs among patents who underwent AE and osteosynthesis was 14.7% (11/75 patients). Among the 11 patients, 6 had Morel-Lavallée lesions, and 2 had open fractures (both involving Faringer zone I) [14]. Nine of the 11 patients with SSIs (81.8%) had undergone nBIIAE. Methicillin-resistant *Staphylococcus aureus* was the most common bacterium causing SSIs (Table 3).

**Table 3:** Demographic details of patients with surgical site infections who underwent AE and osteosynthesis

Case	Sex	Age range (years)	ISS	Associated soft tissue injury	Initial shock	Response to resuscitation	CT findings	Angiography findings and procedure	Embolization material	Surgical approach	Bacteria
1	Male	20–29	35	Morel Lavallée lesion	Yes	Yes	1. CE in left paravesical space 2. Retroperitoneal hematoma	1. Pseudoaneurysm of BIIA, along with pelvic wall hematoma Procedure: BIIAE	Gelfoam	Posterior	MDRAB, E coli., KP
2	Female	30–39	32	None	Yes	No	1. Hematoma over pelvic wall 2. No CE	1. No CE Procedure: BIIAE	Gelfoam	Posterior + anterior	MRSA
3	Male	50–59	32	Open fracture	Yes	No	No CE	1. CE at the anterior division of BIIA Procedure: BIIAE	Gelfoam	Posterior	OSSA
4	Male	20–29	34	Morel Lavallée lesion	Yes	Yes	No CE	1. CE and pseudoaneurysm of left EIA 2. Small pseudoaneurysm at the gluteal region Procedure: BIIAE	Gelfoam	Posterior	MRSA
5	Female	60–69	36	None	Yes	No	Delayed contrast pooling around left retropubic region	No CE Procedure: BIIAE	Gelfoam	Anterior	MRSA
6	Male	20–29	27	Open fracture	Yes	No	CE within the pelvis	1. No CE 2. Tiny pseudoaneurysm at the pelvic floor Procedure: BIIAE	Gelfoam	Anterior	MRSA
7	Female	60–69	17	Morel Lavallée lesion	Yes	No	CE over the RIIA	1. CE at the RIIA Procedure: BIIAE	Gelfoam	Posterior + anterior	E coli
8	Female	40–49	16	Morel Lavallée lesion	Yes	Yes	1. CE at the presacral space 2. Peri-rectal hematoma	1. CE at the LIIA Procedure: BIIAE	Gelfoam	Posterior	E coli
9	Female	40–49	30	Morel Lavallée lesion	No	Yes	CE at the anterior surface of the right hemi-pelvis	CE at the BIIA Procedure: BIIAE	Gelfoam	Posterior + anterior	MRSA
10	Female	18–19	29	Morel Lavallée lesion	Yes	No	CE at the left hemi-pelvis	Abruption termination of the LIIA without CE Procedure: LIIAE	Gelfoam	Posterior + anterior	MSSA
11	Male	40–49	25	None	No	Yes	Hematoma at the left hemi-pelvis	CE at the LIIA Procedure: BIIAE	Gelfoam	Posterior	MSSA

*BIIAE* bilateral internal iliac artery embolization, *CE* contrast extravasation, *CT* computed tomography, *E. coli*: *Escherichia coli*, *EIA* external iliac artery, *ISS* injury severity score, *KP Klebsiella pneumonia*, *LIIAE* left internal iliac artery embolization, *MDRAB* multi drug-resistant *Acinetobacter baumannii*, *MRSA* methicillin-resistant *Staphylococcus aureus*, *MSSA* methicillin-sensitive *Staphylococcus aureus*, *RIIAE* right internal iliac artery embolizatio

Table 4 shows comparisons between patients who did and did not undergo AE. Patients who underwent AE had a higher ISS (26.1 vs. 14.5, *p* = 0.01), a greater likelihood of being in shock upon arrival (68.2% vs. 5.3%, *p* = 0.001), a higher likelihood of having an unstable pelvic fracture pattern (55.9% vs. 19.9%, p = 0.001), and a higher likelihood of undergoing osteosynthesis (57.3% vs. 32.9%, p = 0.001). The AE group also had significantly higher rates of impotence in men (4.8% vs. 0.6%, *p* = 0.036) and SSIs (14.7% vs. 3.7%, *p* = 0.006).

**Table 4:** Comparison of patients who did and did not undergo arterial embolization

	No embolization (*n* = 416)	Arterial embolization (*n* = 129)	*p*-value
Sex, *n* (%)			
Male	205 (49.2%)	62 (48.0%)	0.85
Female	211 (50.8%)	67 (52.0%)	
Mean age, years	45.5 ± 21.1	48.3 ± 22.5	0.25
Shock on arrival, *n* (%)	22 (5.3%)	88 (68.2%)	0.001
Mean ISS	14.5 ± 10.7	26.1 ± 12.0	0.01
Fracture classification, *n* (%) †			
Stable pelvic ring	245 (58.9%)	32 (24.8%)	0.001
Partially unstable pelvic ring	88 (21.2%)	25 (19.3%)	
Completely unstable pelvic ring	83 (19.9%)	72 (55.9%)	
Surgery for pelvic fracture, *n* (%)	136 (32.6%)	75 (58.1%)	0.001
Complications, *n* (%)			
Impotence in men	1 (0.6%)	3 (4.9%)	0.036
Surgical site infection	5 (3.7%)	11 (14.7%)	0.006
Mortality, n (%)	5 (1.2%)	18 (13.7%)	0.001

†The classification of the pelvic fracture was based on the AO/OTA classification (2018 revision)

*ISS* injury severity score

Among the 129 patients who underwent AE, 109 patients (84.5%) had evidence of arterial bleeding at the time of the CT scan. Therefore, the value of AE was evaluated on the basis the characteristics of patients with and without identifiable CE during CT (Table 5). No significant inter-group differences were detected in initial shock status, response to resuscitation, age, sex, ISS, time to AE, fracture classification, volume of blood transfusion, or rate of repeat AE. However, patients without identifiable CE during CT or angiography had a significantly higher 1-week in-hospital mortality rate than patients with identifiable CE (30.0% vs. 11.0%, *p* = 0.03).

**Table 5:** Comparison of patients who underwent arterial embolization with and without evidence of contrast extravasation during computed tomography

	Evidence of CE (*n* = 109)	No evidence of CE (*n* = 20)	*p*-value
Sex, *n* (%)			0.47
Male	54 (49.5%)	8 (40.0%)	
Female	55 (50.5%)	12 (60.0%)	
Mean age, years	48.1 ± 22.9	50.1 ± 20.7	0.34
Mean ISS	26.2 ± 12.5	28.4 ± 9.8	0.13
Time from order to angiography, min	64.6 ± 47.2	62.9 ± 30.7	0.67
Repeat AE, n (%)	3 (2.8%)	2 (10.0%)	0.17
Fracture classification, n (%)			0.68
Stable ring	30 (27.5%)	6 (30.0%)	
Partially stable ring	32 (29.3%)	4 (20.0%)	
Unstable ring	47 (43.2%)	10 (50.0%)	
Mean blood transfusion (mL)	2348.1 ± 2727.0	2606.0 ± 2097.2	0.25
Embolization material			0.23
Gelfoam	101(92.7%)	17(85.0%)	
Metal coils	8(7.3%)	3(15.0%)	
Osteosynthesis for pelvic fracture, n (%)	62 (56.9%)	13 (65.0%)	0.32
Embolization of BIIA, n (%)	82 (75.2%)	15 (75.0%)	0.59
Mortality, n (%)	12 (11.0%)	6 (30.0%)	0.03

*CE* contrast extravasation, *ISS* injury severity score, *AE* arterial embolization, *BIIA* bilateral internal iliac arteries

One of the indications for AE in our institution was the presence of CE during CT. Therefore, we examined the relationship between identifiable bleeding during CT or during angiography (the standard for comparison) to evaluate the effectiveness of CT for assessing arterial bleeding in patients with unstable pelvic fractures. We used the angiography results as a standard baseline to examine the predictive value of CT examination. All 131 patients who underwent angiography had undergone CT examination upon their emergency department arrival. The CT examination revealed retroperitoneal bleeding in 108/131 patients (82.4%), although CE during angiography was only identified in 34/131 patients (26%). Among the 34 patients with CE during angiography, 32 patients (94.2%) had CE during the CT examination. Predictive analysis revealed that CE during CT had a sensitivity of 94.1%, a specificity of 21.6%, a positive predictive value (PPV) of 29.6%, and a negative predictive value (NPV) of 91.3%.

## Discussion

Pelvic fractures may be accompanied by hemorrhage, which can lead to hemodynamic instability and death. At our institution, AE intervention is a standard procedure in this setting and is associated with good bleeding control outcomes [8, 10]. Furthermore, delayed angiography in these patients may be associated with poor outcomes [8, 15, 16]. Technological improvements and increased availability have led to an increasing number of vascular injuries being identified during CT examinations, although recent studies have indicated that imaging-based evidence of CE does not necessarily indicate a need for AE to control bleeding [4, 17–19]. Thus, hasty AE following pelvic fracture might not be an optimal step during the busy resuscitation process. Although AE can be promptly performed at our center (in a mean time of 60 min from order to procedure), the main diagnostic tool was CT, which had a low PPV, and the high incidence of SSIs following nBIIAE was suboptimal. For example, 82.4% of patients in the AE group exhibited signs of arterial bleeding during the CT examination, while only 26% of patients exhibited signs of CE during angiography. This corresponds to a PPV of only 29.6% for the use of CT to detect arterial injury in patients with pelvic fractures, which is similar to the results from previous studies [13, 20]. We suspect that it may be difficult to differentiate between bone marrow and arterioles as the source of contrast extravasation during CT examination soon after a pelvic fracture, and our results suggest that evidence of CE during CT may not be a suitable major indication for AE, despite this being previously recommended [7, 21]. Dreizin et al. [22] have suggested that the use of AE should be guided instead by the hematoma volume when arterial bleeding is detected during CT. We believe that angiography and AE should be performed more selectively, perhaps on the basis of non-response to resuscitation, a continuing decrease in hemoglobin concentration without evidence of bleeding from other sources, and the absence of arterial blush during CT.

Non-selective embolization is considered a hemostatic procedure for hemodynamically unstable patients with pelvic fractures [7, 23], although recent studies have focused on selective or super-selective embolization to decrease the negative effects of non-selective embolization in cases of vasospasm or rich collateral arterial networks within the pelvis [24, 25]. Thus, we evaluated the outcomes of different AE strategies, given the fact that AE interventions might be accompanied by a high complication rate after these patients undergo osteosynthesis to treat their unstable pelvis. The present study revealed that 74% of patients underwent nBIIAE as their AE intervention, and 9 of 11 patients who developed SSIs after AE and osteosynthesis had undergone nBIIAE. Local soft tissue injuries after high-energy trauma, such as Morel-Lavallée lesions, might have contributed to the incidence of SSIs, although it would also be prudent to consider the potential contribution of nBIIAE.

Contrast-enhanced CT scans have become routine at many institutions, including ours, to detect arterial bleeding within the pelvis [26]. However, relative to angiography as the gold standard, CT had a low PPV and a high NPV for detecting arterial bleeding. In addition, we observed that patients without signs of CE during imaging had a significantly higher mortality rate after AE, relative to patients with signs of CE during CT (30.0% vs. 11.0%, *p* = 0.03). Moreover, there were no significant differences between the two groups in age, sex, ISS, time to AE, fracture classification, volume of blood transfusion, or rate of repeat AE. We speculate that patients without signs of CE during CT would have been experiencing shock due to causes other than arterial injury, such as cancellous fracture surface bleeding or venous plexus bleeding. As a result, AE may have provided little benefit in these patients, while delaying the resuscitation procedure and potentially increasing the risk of mortality. Therefore, AE should not be a routine procedure for hemodynamically unstable patients with unstable pelvic fractures, and other hemostasis procedures, such as PPP, should be considered during the resuscitation process for these patients.

Although we made every effort to reduce the influence of bias in this study, it has several limitations. First, the retrospective design is inherently associated with risks of bias, and we observed that cases that underwent AE were more severe and had higher ISS scores than the non-AE cases. Thus, the higher complication rate in the AE group might reflect the severity of the patients’ injuries, and strict selection criteria may be needed for the AE procedure to avoid further complications that are difficult to manage. Second, although our center uses an established protocol to identify patients who should undergo angiography, the choice of selective or non-selective embolization was at the radiologist’s discretion. The interventional radiologists commonly noted that non-selective embolization was performed “to facilitate hemostasis,” even when no arterial bleeding was identified during angiography. Third, AE has been performed at our center during the last two decades as part of a fixed protocol for hemodynamically unstable patients with pelvic fractures, and it may be difficult to encourage experienced emergency traumatologists to include PPP as an alternative strategy in our resuscitation protocol.

## Conclusions

In conclusion, we believe that angioembolization is an effective, time-saving, and readily available procedure for pelvic fracture-related arterial hemorrhage. However, given the high rate of SSIs, non-selective AE may not be the best practice in this setting when there is no sign of CE during CT or when the patient does not respond to resuscitation after other sources of hemorrhage have been excluded. It may be appropriate to perform more selective or super-selective embolization procedures in carefully selected patients and to consider PPP as an alternative hemostasis strategy in the resuscitation protocol when there are no imaging-based signs of arterial bleeding.

## Data Availability

All data generated and analyzed during this study are included in the published article.

## Abbreviations

AE: Arterial embolization
CE: Contrast extravasation
CT: Computed tomography
ED: Emergency department
ISS: Injury severity score
NPV: Negative predictive value
nBIIAE: Nonselective bilateral internal iliac artery embolization
SSI: Surgical site infection
PPP: Preperitoneal pelvic packing
PPV: positive predictive value
SSI: Surgical site infection
